# Continuous caloric restriction and diet replacement but not alternate-day fasting may enhance susceptibility to glucose intolerance, dyslipidemia and cell stress under renewed high-fat diet

**DOI:** 10.1007/s40200-026-02112-1

**Published:** 2026-10-21

**Authors:** Andrea Cristiani Beuren, Jaíne Borges dos Santos, Natascha Fill Izolan, Lílian Corrêa Costa-Beber, Lucas Machado Sulzbacher, Matias Nunes Frizzo, Pauline Brendler Goettems-Fiorin, Thiago Gomes Heck, Mirna Stela Ludwig

**Affiliations:** 1 Graduate Program in Integral Health Care (PPGAIS/UNICRUZ/URI-Erechim/UNIJUÍ), Regional University of Northwestern Rio Grande do Sul State (UNIJUÍ), Ijuí, RS Brazil; 2 Brigham and Women’s Hospital, Boston, MA USA; 3 Graduate Program in Mathematical and Computational Modeling (PPGMMC), Regional University of Northwestern Rio Grande do Sul State (UNIJUÍ), Ijuí, RS Brazil; 4 National Institute of Science and Technology - INCT Physical (In)activity and Health, CNPq, Brazil; 5 Universidade Regional do Noroeste do Estado do Rio Grande do Sul (UNIJUI), Bairro Universitário, Rua do Comercio, 3000 – Bairro Universitário, Ijui, 98700-000 Rio Grande do Sul Brazil

**Keywords:** Dietary interventions, Body weight fluctuations, Oxidative stress, Dyslipidemia

## Abstract

**Background:**

Weight regain affects most individuals attempting to treat obesity and its comorbidities, and it is associated with increased risk of metabolic and cardiovascular diseases. Several strategies have been proposed to induce weight loss, including caloric restriction (CCR), diet replacement (DR) and alternate-day fasting (ADF).

**Aim:**

To compare these approaches in terms of weight maintenance and metabolic, oxidative, and inflammatory outcomes.

**Methods:**

Male Wistar rats underwent a three-phase protocol. First, animals received a high-fat diet (HFD) for eight weeks to induce obesity. Second, they were divided into four groups: *ad lib*-HFD (continued HFD), R-HFD (30% reduction of HFD w/w), ADF-HFD (24 h fasting alternating with 24 h *ad libitum* HFD), and *ad lib-*LFD (DR via *ad libitum* access to low-fat chow) for six weeks. Finally, all groups returned to free access to HFD for four weeks.

**Results:**

All interventions delayed weight gain; however, discontinuation led to rapid weight regain. Under renewed HFD *ad libitum* exposure, CCR and DR induced higher susceptibility to dyslipidemia, as shown by the temporal analysis. These strategies also impaired hepatic and adipose antioxidant and anti-inflammatory responses and heightened lymph node stress upon return to unlimited offer of obesogenic diet. In contrast, ADF appeared to confer partial protection against the metabolic complications of weight rebound.

**Conclusions:**

Despite similar weight-loss efficacy, ADF may better preserve metabolic homeostasis during weight regain.

**Supplementary Information:**

The online version contains supplementary material available at https://doi.org/10.1007/s40200-026-02112-1.

## Introduction

Obesity is a chronic disease and one of the most pressing public health challenges worldwide, with its prevalence more than doubling since 1990 and now affecting one in every eight individuals [1]. It is characterized by multiple metabolic impairments, including insulin resistance and dyslipidemia, which contribute to a 22% and 49% higher risk of all-cause and cardiovascular mortality, respectively [2]. In contrast, sustained weight loss improves clinically relevant metabolic and cardiovascular markers, such as hemoglobin A1c and total cholesterol levels, and remains the first-line intervention for obesity and its complications [3].

Several strategies have been proposed to induce weight loss. Continuous caloric restriction (CCR) is the standard approach [3], achieved either through moderate reduction of total energy intake without significantly changing the quality of the usual diet, or through macronutrient substitution, such as lowering dietary lipids [4]. However, CCR is often difficult to maintain due to the constant need to regulate both food quantity and quality [5]. In this context, intermittent fasting (IF) emerged as an alternative that preserves food composition while alternating periods of feeding and fasting. IF comprises alternate day fasting (ADF), marked by *ad libitum* consumption for 24 h, followed by 24 h of fasting, short-term fasting, involving limiting daily energy intake to approximately 25% on either 2–3 consecutive or non-consecutive days of the week, and time-restricted eating, characterized by consuming within a daily window of less than 12 h [6, 7]. Among these, ADF is the easiest to adapt to animal models, without interfering with their circadian rhythms and adding this bias on the metabolic outcomes [8]. Previous studies demonstrated that ADF produces weight loss comparable to CCR [6, 9] and may confer superior metabolic and inflammatory benefits [10]. Additionally, ADF has shown to downregulate genes related to muscle atrophy during fasting, which could help in alleviating overall inflammatory scenario and adipose tissue dysfunctionality during weight loss [10]. Nonetheless, evidence comparing these approaches regarding the post-interventional period, weight maintenance and metabolic outcomes are scarce.

The adaptation period following weight loss is critical. Nearly half of individuals regain all lost weight within 12 months, and only 11% maintain a 5-kg reduction after five years [11], largely due to the resumption of previous habits. Recurrent cycles of weight loss and regain, known as “weight cycling” or “yo-yo dieting,” have been associated with increased risk of diabetes and adverse cardiovascular outcomes, sometimes exceeding the risk observed in stable obesity [12, 13]. Key metabolic tissues, including liver, skeletal muscle, and white adipose tissue (WAT), are particularly vulnerable during weight cycling. Evidence indicates the occurrence of adipocyte hypertrophy [14, 15], compensatory lipogenesis [15] and impaired redox and mitochondrial function in skeletal muscle, that together result in lower resting energy expenditure [16] and worse cardiometabolic risk. Thus, identifying weight-loss strategies that minimize rebound and its metabolic consequences is a major clinical need.

Weight oscillations and metabolic dysfunctions converge on alterations in the cellular heat shock response (HSR), particularly through modulation of 70 kDa-heat shock proteins (HSP70). Intracellular HSP70 (iHSP70) acts as a molecular chaperone with potent anti-inflammatory and antioxidant functions. It supports superoxide dismutase (SOD) maturation [17], inhibits nuclear factor κB (NF-κB) chronic activation [18], and is determinant in cell survival [19]. Under stress, HSP70 may be released extracellularly (eHSP70), where it functions as a damage-associated molecular pattern (DAMP) that activates leukocytes and amplifies inflammation [19, 20]. Impaired HSR is linked to cellular senescence [21, 22], low-grade inflammation, and the progression of metabolic and cardiovascular diseases [23–25].

Our group has consistently shown that high-fat diet (HFD)–induced obesity progressively disrupts HSR, thereby exacerbating metabolic dysfunction and inflammation [26–28]. Building on this evidence, we aimed to compare different weight-loss strategies, ADF, CCR, and DR, regarding their effects on weight regain and on metabolic and cellular responses in male rats. We hypothesized that ADF would confer partial protection against the metabolic dysfunction associated with weight rebound.

## Methods

### Ethical considerations

This study followed all ethical rules established by Arouca’s Act (Federal Law 11794/2008) and the Guide for Care and Use of Experimental Animals, published by the National Institutes of Health (NIH publication no. 85–23, revised in 1996). This protocol was approved by the Animal Ethics Committee of UNIJUI (CEUA 005/19).

### Experimental design

Male Wistar rats, eight weeks old (*n* = 32, approximately 233 ± 32 g), were obtained from the Animal Facility of the Regional University of the Northwestern Rio Grande do Sul State (UNIJUI). They were kept in specific boxes for rodents, 2 to 3 rats per cage, with a bottom lined with wood shavings, under controlled conditions of temperature (22 ± 2 °C), light-dark cycles (light from 7:00 a.m. to 7:00 p.m.) and free access to water.

The study was conducted in three main phases. In the first phase, all animals (*n* = 32) received high-fat diet (HFD) for eight weeks, to induce obesity and glucose intolerance [29–31]. In the second phase, animals were randomly divided into four experimental groups, for six weeks, according to the adopted dietary regimen. Body weight was checked and statistically compared to ensure uniformity. *Ad lib*-HFD group (*ad lib*-HFD, *n* = 8) was maintained on HFD *ad libitum* and had their food intake recorded and used as a reference for the group undergoing CCR. CCR can be achieved by changing food quality [32], and reducing the caloric intake relative to the usual diet [33], both ameliorating metabolic profile in rats [33]. In this sense, as we worked with HFD as a background to induce obesity and glucose intolerant model, we established CCR relative to *ad libitum* consumption of HFD. Thus, animals submitted to CCR (R-HFD, *n* = 9) received 30% less HFD (w/w) compared to the *ad lib*-HFD group [34–36]. Also, animals undergoing ADF (ADF-HFD, *n* = 9) had HFD *ad libitum* for 24 h, followed by 24 h of fasting. Since we understand that diet quality is a concern for weight loss, we adopted diet replacement DR (*ad lib*-LFD, *n* = 6) as the fourth experimental group, which received regular low-fat diet (LFD) *ad libitum* [37–40]. Interestingly, hyperphagia on the *ad lib*-HFD group relative to *ad lib*-LFD group has not been confirmed by our research group [29]. In the end of the second phase, blood was collected through tail veil to measure cholesterol and triglycerides. Additionally, glucose tolerance test (GTT) was done in the baseline, end of first, second and third phases. Finally, in the last phase, defined as weight maintenance, all the animals received HFD *ad libitum* for four weeks. Then, animals were killed, tissues and whole blood collected.

Sample size calculation was done using previous studies (Table S1), considering *P* < 0.05. Based on Higashida et al. [37], a large effect size was estimated for body weight changes (Cohen’s d ≈ 1.6). Sample size calculation (α = 5%; power = 80%) indicated a minimum of 7 animals per group. Thus, 8–9 animals per group were initially included to account for experimental losses. One group completed the protocol with 6 animals due to losses inherent to the experimental model, without substantial impact on the statistical robustness of the study.

**Fig. 1:**
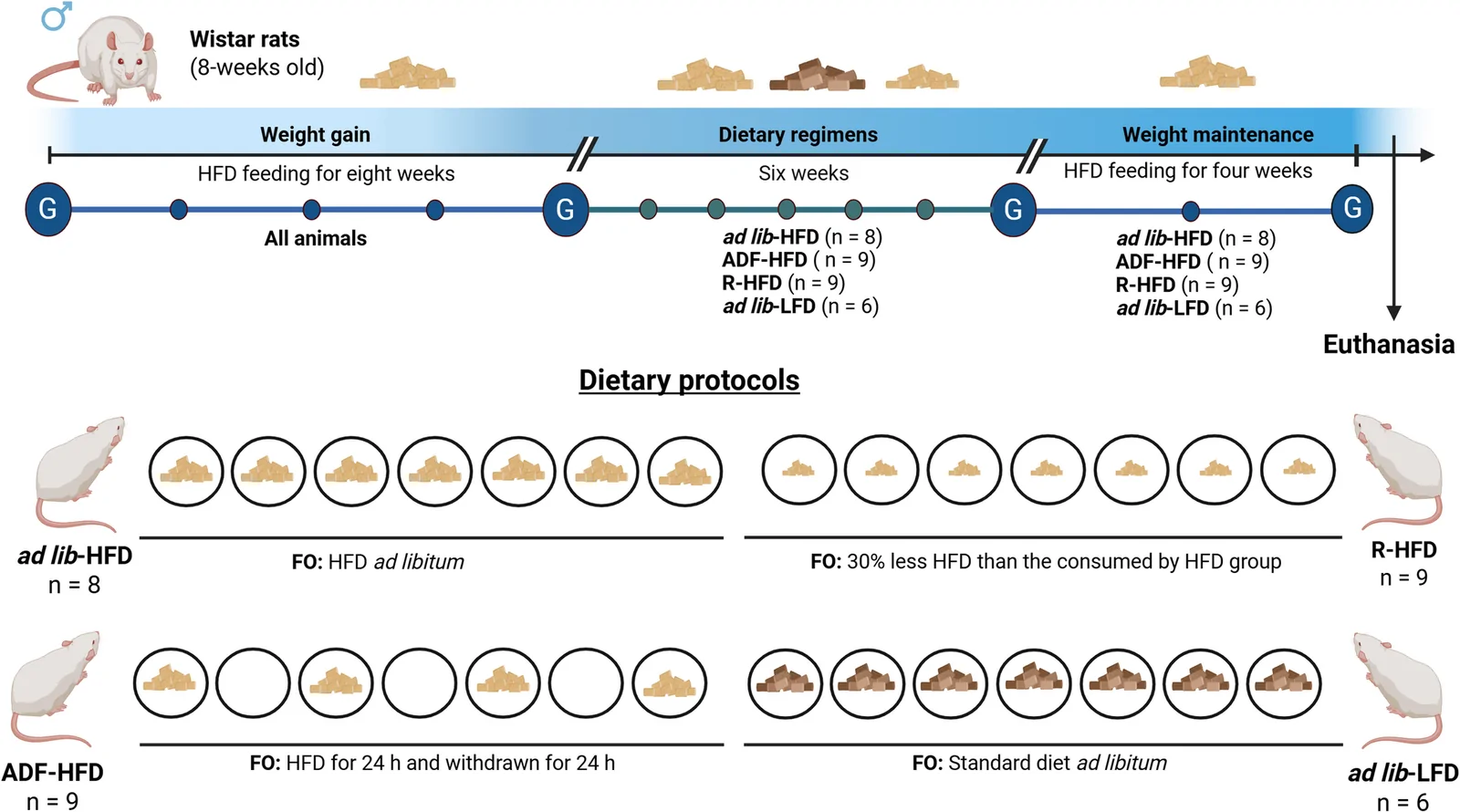
Experimental design. In the first phase, animals were fed a high-fat diet (HFD) for eight weeks to induce obesity and insulin resistance. Once the model was established, they were allocated into four experimental groups (*ad lib*-HFD, R-HFD, ADF-HFD, and *ad lib*-LFD) and maintained on their respective dietary regimens for six weeks. The *ad lib*-HFD group (*n* = 8) remained on HFD *ad libitum*, and its recorded food intake served as the reference for the continuous caloric restriction group (R-HFD, *n* = 9), which received 30% less HFD (w/w). The alternate-day fasting group (ADF-HFD, *n* = 9) alternated 24 h of *ad libitum* HFD with 24 h of fasting, while the substitution diet group (*ad lib*-LFD, *n* = 6) received regular chow *ad libitum*. In the final phase, defined as weight maintenance, all animals returned to *ad libitum* HFD for four weeks. Body weight and Lee Index were measured every two weeks during phases 1 and 3, and weekly during phase 2. Glucose tolerance tests (GTT) were performed at the beginning and end of each phase. At completion of the study, animals were euthanized, and blood and tissues were collected for further analysis. FO: food order. This layout was created in https://BioRender.com

### Diets and intake

#### Diets

The used HFD is a lard-based hyperlipidemic diet (37.4% w/w), with total metabolizable energy of 22.8 MJ/kg (58.3% as fats, 24.5% as carbohydrates, and 17.2% as proteins). All ingredients (except for starch and lard) were adjusted to be present in the same amount as in the standard rodent diet. To meet these requirements, 13.7% egg albumin, 7.4% amino acid supplement (Aminomix™), and 1.1% dried bone and oyster flour were used to prepare the experimental diet fresh weekly [27–30, 41]. The HFD was prepared weekly in the Laboratory of Biological Assays (UNIJUI) and was refrigerated (4 ◦ C–8 ◦ C) during this period. On the other hand, the regular chow offered to the *ad lib*-LFD group was commercially obtained from Nuvital Nutrientes SA. (Nuvilab CR-1) and had a total metabolizable energy of 16.6 MJ/kg, 11.4% as fats, 62.8% as carbohydrates, and 25.8% as proteins.

#### Food intake

The food consumption was calculated as the difference between the amount provided and the residuals left in the cages and normalized by the number of animals [(offered – remaining) / nº of animals in the cage]. This value was then used to determine daily energy consumption (kcal/d) by multiplying by the energetic density of each diet (22.8 MJ/kg and 16.6 MJ/kg for HFD or LFD, respectively) and normalizing by the number of days between food offer and intake calculation: [(offered – remaining) x (energetic density)] / [(nº of animals) x (nº of days)] [34]. There was no obvious spillage, judged by looking, on any day. This parameter was checked thrice a week, except during the second phase, when it was performed daily. Thus, every cage had three or seven measurements per week (average consumption for animals per day), which were considered as individual “n” to do the statistics. All procedures were conducted after 7:00 p.m.

### Biometrical parameters

Body weight, and naso-anal length (NAL) were measured every two weeks in the first and third phases, and weekly in the second phase (Fig. 1). These numbers were used as a basis to calculate the Lee Index, which is a murine equivalent for body mass index (BMI) (Lee, 1929), according to the formula [42]: Lee index = [(∛(Weight(g))/ (NAL (cm)) x 1000].

Also, at the end of the experimental design, the estimated adiposity was calculated by the percentage of epididymal WAT related to total body weight [(% eWAT/ body weight) x100] [27–29].

### Glucose Tolerance Test (GTT)

We performed GTT at the baseline and at the end of first phase, to confirm the HFD-induced glucose intolerance, immediately after the second and 48 h before the end of the third phase, to follow the effects of the dietary interventions (Fig. 1). For this, food was withdrawn overnight and GTT was conducted after 12 h of food deprivation. Glycemia was measured immediately before and at 15, 30, and 120 min after 80% glucose (1 g/kg in saline solution, *i.p.*) administration, in the total blood from the caudal vein using the *Accu-Chek*^*®*^*Active* glucometer. The glycemic response during GTT was evaluated by the AUC method [27].

### Euthanasia and tissues collection

At the end of the experimental design (after third phase), after eight hours of food deprivation, the animals were killed through decapitation, without anesthesia. The decapitation method was chosen because it is rapid and does not alter glycemia [43, 44] or other inflammatory parameters, that could result in fluctuations in HSP70 [45], whose expression represents a critical variable in our study. The entire procedure was performed in an exclusive environment with exhaustion, and entire hygiene with 70% ethanol was made after each animal.

Immediately after the euthanasia, blood was collected and put into a tube containing ethylenediaminotetra-acetic acid (EDTA) (2 mg/mL), or clot activator. Both were centrifuged at 3000 rpm, for 15 min, for plasma and serum obtaining, respectively. Serum was destined to lipids, glycaemia and insulinemia dosages, and plasma to oxidative stress measurements. The eWAT, gastrocnemius muscle, liver and mesenteric lymph nodes were collected, weighted and frozen in liquid nitrogen for further molecular analysis. Part of the liver was put into fixative solution [4% paraformaldehyde (PFA)] for histological analysis.

### Blood analysis

#### Blood cells count

The blood was conditioned in a tube containing the anticoagulant EDTA (5 µL of EDTA per 500 µL of blood) to determine hematological parameters through the automated hematology analyzer (ABX, Horiba). Neutrophils to lymphocytes (NLR), monocytes to lymphocytes (MLR) and platelets to lymphocytes ratio (PLR) were calculated using the absolute number of the corresponding cells. The systematic inflammation response index (SIRI) was calculated using the following formula: SIRI = (Neutrophils × Monocytes) / Lymphocytes [46].

#### Biochemistry

Insulin was measured through the electrochemiluminescence method, and data was expressed as µU/mL. Glycaemia, triglycerides, total cholesterol and HDL-C were measured by Bioclin-Quibase colorimetric kits. LDL-C was calculated through the Friedewald formula $$\:LDL=total\:cholesterol-HDL-\raisebox{1ex}{$TG$}\!\left/\:\!\raisebox{-1ex}{$5$}\right.$$ [47]. Data was expressed as mg/dL.

Aspartate aminotransferase (AST), alanine aminotransferase (ALT) and gamma-glutamyl transferase (GGT) were measured in serum samples using Bioclin Quibasa kits and the automated BS200 system (as above), by kinetic methods. Results were expressed in U/L.

### Liver composition and morphological analysis

#### Cholesterol and triglycerides content

For the analysis of the lipid content, tissues were homogenized in isopropanol (50 mg/mL). Liver samples were centrifuged at 3136 *x* g for 10 min and the supernatant collected. For triglycerides and cholesterol analysis, 10 µL and 20 µL of homogenate were incubated with 500 µL of reagent (Labtest^®^), respectively. The results were expressed as mg cholesterol/g of tissue, and % of triglycerides relative to the liver.

#### Histology

After the removal, three portions of the tissue were immediately put into 20-fold volume of fixative solution (4% PFA), in histological cassettes with removable lids and automatically processed by Lupetec PT 05^®^. Later, tissues were included in metallic molds, in warmed plate, and embedded in paraffin. Blocks were sectioned using a microtome (5 μm section thick), deparaffined, rehydrated, and stained with hematoxylin-eosin according to standard procedures [20, 48].

Transverse sections of the liver were observed with light microscopy at a final magnification of 400x (Quimis binocular microscope, Academic Q709TK-PL model), and analyzed for the presence, quantity, and presentation of steatosis, hepatocellular ballooning (defined as enlarged hepatocytes that were distinguishable from background hepatocytes and exhibited partially clear or altered cytoplasm), and fibrous tissue within the observed field. In addition, the cellular morphology of the randomly selected field was assessed. Other documented features included the types of steatosis (macrovesicular, microvesicular, or mixed).

The samples were then classified according to the severity of fat accumulation and the morphology of the hepatocellular ballooning observed. Brunt’s method was chosen, which evaluates steatosis based on the general impression of the histological sections, presence of inflammation and hepatocellular balloon [49]. Then, its gravity was categorized as low (G1), moderate (G2) and high (G3). All analysis was performed thrice and measured blindly.

### Molecular and oxidative stress analysis

#### Tissue preparation

Liver, gastrocnemius muscle, eWAT and mesenteric lymph nodes were homogenized in potassium phosphate buffer (KPi, pH 7.4) and 0.1% sodium dodecyl sulfate (SDS) plus proteases inhibitor [phenylmethylsulfonyl fluoride (PMSF, 100 µM)] for oxidative stress and western blotting analyses, respectively. Then, they were centrifuged at 1,254.4 *x* g, for 10 min and the supernatant divided into different tubes for each technique.

#### **Lipoperoxidation**

Oxidative damage to the lipids was evaluated through the lipoperoxidation levels, obtained via the thiobarbituric acid (TBA) reactive substances (TBARS) test [50], using a standard curve with 1,1,3,3-tetramethoxypropane, ranging from 0.0005 to 0.016 mg/mL. Homogenates (100 µL) were incubated with equivolume of 10% trichloroacetic acid (TCA) in ice for 30 min and centrifuged. The supernatant was incubated with 0.67% TBA (80 µL) at 95 °C, for 15 min. The absorbances were verified at 535 nm.

#### Antioxidant enzymes activity

SOD activity was assessed by the inhibition of pyrogallol auto-oxidation [51], at 420 nm. For this, the samples were incubated with 50 mM Tris- 1 mM EDTA buffer (pH 8.2), and 30 µM catalase in the spectrophotometer, at 37 ◦C. After homogenizing, 24 mM pyrogallol (in 10 mM HCl) was added to the mix. The absorbances were verified every 20 s, during 120 s. The results were expressed as unit of SOD (USOD)/mg prot.

Catalase activity was determined via H_2_O_2_ decomposition [52], at 240 nm. For this, the samples were incubated with 50 mM phosphate buffer (pH 7.4) in the spectrophotometer, at 32 ◦C. After homogenizing, 0.01 M H_2_O_2_ was added to the mixture. The absorbances were verified every 15 s, during 120 s and the results expressed as unit of CAT (UCAT)/mg prot. The total protein concentration was spectrophotometrically verified, using the method of Bradford [53], at 595 nm.

#### HSP72 levels

HSP70 was measured in plasma, liver, gastrocnemius and eWAT using a Kit AMP’D HSP72 high sensitivity enzyme-linked immunosorbent assay (ELISA) [Enzo, EKS-715, 7 pg/mL (range 0.039–5 ng/mL)]. The solid tissues were homogenized in assay buffer, in the 1:20 proportion, using 1 µL of proteases inhibitor per sample (PMSF). Then, they were centrifuged at 4000 rpm, for 10 min. The supernatants were collected and diluted (1:1 in assay buffer), to further incubation in the antibody-loaded plate. The assay was carried out according to the manufacturer’s specifications. The absorbance was verified at 495 nm, and results expressed as ng HSP70/g of tissue.

#### Western blotting

iHSP70 levels in lymph nodes were assessed by western blotting. Approximately 40 µg of protein from each sample was mixed with sample buffer (50 mM Tris, 10% SDS, 10% glycerol, 10% 2‑mercaptoethanol, and 2 mg/mL bromophenol blue). Samples were boiled for 10 min and separated on a 10% polyacrylamide gel for 5 h at 15 mA per gel. Proteins were then transferred onto a nitrocellulose membrane (GE Healthcare) using electrotransfer for 1 h at 100 V. Transferred bands were visualized with 0.3% (w/v) Ponceau S (Sigma‑Aldrich).

Membranes were rinsed with washing buffer (TEN–Tween 20 [0.1% w/v]: 50 mM Tris, 5 mM EDTA, 150 mM NaCl, pH 7.4) and blocked with 5% (w/v) nonfat dry milk prepared in the same buffer. They were incubated for 12 h with a monoclonal anti‑HSP70 antibody (Sigma‑Aldrich H5147, 1:1000). After three washes, membranes were incubated with peroxidase‑conjugated rabbit anti‑mouse IgG (Sigma‑Aldrich A9044, Lot #018M4899V, 1:15,000) for 1 h, followed by three additional washes. Peroxidase‑labeled anti‑actin (Sigma‑Aldrich A3854, Lot #048M4859, 1:50,000) was used as a loading control.

Blots were developed using ECL‑Prime western blotting reagent (GE Healthcare). Band intensities were quantified with ImageJ^®^ software. Results are expressed as arbitrary units of iHSP70, normalized to β‑actin and to the lowest value on the gel as well as the control mean.

### Statistical analysis

The distribution of all variables was first assessed using the Kolmogorov–Smirnov test. Parametric data were analyzed by One-way ANOVA followed by Tukey’s for multiple comparisons, unless stated otherwise. Model validation was performed by comparing body weight and Lee-index at baseline and after 8 weeks of HFD consumption using paired T-test. GTT-AUC between these two phases was evaluated by One-way ANOVA with repeated measures followed by Tukey’s test.

Comparisons across the second and third phases, including body weight, Lee-index, GTT curves, GTT-AUC, plasma total cholesterol, and triglyceride levels, were conducted using Two-way ANOVA with repeated measures followed by Šídák’s test. Non-parametric variables (insulin levels and steatosis severity) were analyzed using Kruskal–Wallis.

Liver, eWAT, gastrocnemius iHSP70, eHSP70, gastrocnemius CAT, and lipoperoxidation, liver SOD and lipoperoxidation were transformed to meet parametric assumptions using square-root transformation and analyzed by One-way ANOVA followed by Tukey. Pearson’s correlations were applied when appropriate.

All analyses and graphical representations were performed in GraphPad Prism 10, with statistical significance set at *P* < 0.05. Significant group differences identified by Tukey’s post-test are indicated in the figures.

## Results

### Dietary interventions delay weight gain, but enhance the susceptibility to weight rebound under free access to obesogenic diet

**Fig. 2:**
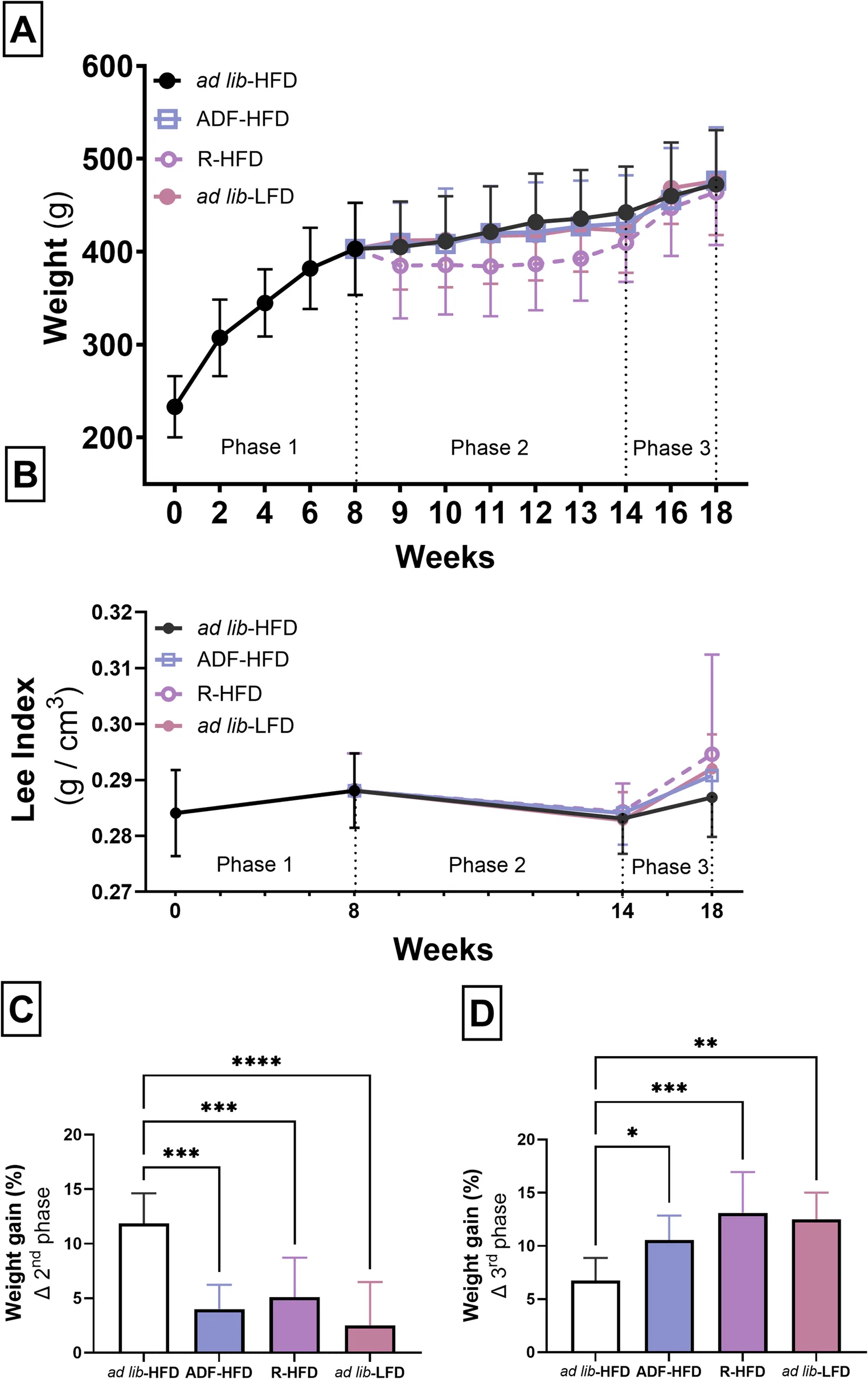
Dietary interventions delay weight gain, but the gain returns to normal on obesogenic diet. (**A**) Body weight during the experiment. Paired analysis identified that *ad lib*-HFD group gained weight in the first phase (Paired t-test: *P* < 0.0001), *ad lib*-HFD and ADF-HFD gained weight in the second phase (RM Two-way ANOVA: Time: F _2.928, 81.97_ = 58.04, *P* < 0.0001, Intervention: F _3, 28_= 0.686, *P* = 0.568; Time vs. intervention: F _8.783, 81.97_ = 8.856, *P* < 0.0001), and that all groups gained weight in the last phase (RM Two-way ANOVA: Time: F _1.803_, _50.48_ = 107.3, *P* < 0.0001, Intervention: F _3, 28_ = 0.205, *P* = 0.891, Time vs. intervention: F _5.409, 50.48_ = 2.421, *P* = 0.044). No differences among groups were identified. (**B**) Lee Index during the experiment. Paired analysis identified that *ad lib*-HFD group increased the Lee Index in the first phase (Paired t-test, *P* = 0.001), *ad lib*-HFD, ADF-HFD and *ad lib*-LFD reduced this index in the second phase (RM Two-way ANOVA: Time: F _1, 28_ = 50.25, *P* < 0.0001, Intervention: F _3, 28_ = 0.058, *P* = 0.980, Time vs. intervention: F _3, 28_ = 2.859, *P* = 0.054) and R-HFD enhanced it in the last phase (RM Two-way ANOVA: Time: F _1.386, 38.80_ = 16.91, *P* < 0.0001, Intervention: F _3, 28_ = 0,203, *P* = 0.892, Time vs. intervention: F _4.158, 38.80_ = 0.729, *P* = 0.582). No differences among groups were identified. **C-D.** Relative weight gain (%) in the second phase (Δ14 − 8) (One way ANOVA: F _3, 28_ = 13.22, *P* < 0.0001) (**C**) and the third phase (Δ18 − 14) (One way ANOVA: F _3, 28_ = 8.268, *P* = 0.0004) (**D**). Data expressed as mean ± SD. *P < 0.05, ** *P* < 0.01, *** *P* < 0.001 and *** *P* < 0.0001 represent differences between experimental groups (Tukey’s post-test)

Two months of HFD feeding increased body weight (Fig. 2A) and Lee Index (Fig. 2B), a reliable, non-invasive method to infer adiposity in experimental models as it correlates with body fat [42]. Therefore, the increase in this index, a rodent analogue of BMI [54], and the impaired glucose tolerance (Figure S1A-B) during the first phase of our experimental design confirmed the development of obesity. Additionally, our lab has consistently shown in rodents of both genders and in male rats that this HFD enhances body adiposity and impairs glycemic control after short-term consumption (4 weeks) [27, 29, 31] (Figure S2A-E). Moreover, a recent manuscript published by our research group reported that 8 weeks-HFD consumption prior to the interventions is effective to induce the obesity model [55].

Between weeks 8 and 14, only the *ad lib*-HFD and ADF-HFD group showed significant weight gain in paired analyses (Fig. 2A). Although all dietary interventions slowed weight gain during the second phase (Fig. 2C), each resulted in greater weight gain during the maintenance/regain (third) phase compared with animals fed HFD *ad libitum* (*ad lib*-HFD group) (Fig. 2D). Paired analyses further revealed that all groups except R-HFD reduced their Lee Index. The reduction observed in the *ad lib*-HFD group was attributable to increased body length (Figure S3), which is inversely related to the index. Notably, between weeks 14 and 18, R-HFD was the only group to exhibit an increase in the Lee Index (Fig. 2B). The groups did not differ in the final weight, Lee Index, estimated adiposity or liver mass (Table 1).

**Table 1:** Dietary interventions did not interfere with the liver index nor estimated adiposity

	ad lib-HFD	ADF-HFD	*R*-HFD	ad lib-LFD	One-way ANOVA
eWAT (%)	2.59 ± 0.9	2.62 ± 0.5	2.75 ± 0.8	2.96 ± 0.7	F_3, 28_ = 0.346, *P* = 0.791
Liver mass (%)	3.082 ± 0.256	2.989 ± 0.225	3.080 ± 0.254	3.061 ± 0.057	F_3, 28_ = 0.339, *P* = 0.797

Note: *eWAT* epididymal white adipose tissue. Data expressed as mean ± SD

The benefits observed on weight gain were compatible with the lower estimated energetic intake (Figure S4). Interestingly, in the third phase, when HFD was offered *ad libitum*, the ADF-HFD and the R-HFD still had the lowest energetic intake, with the last one presenting the minimal area under the curve (AUC) (Figure S4B-C). It was not observed in the *ad lib*-LFD group, whose average consumed was higher than all other groups (Figure S4B-C).

### Prior CCR and DR predispose animals to metabolic impairment under free access to obesogenic diet

**Fig. 3:**
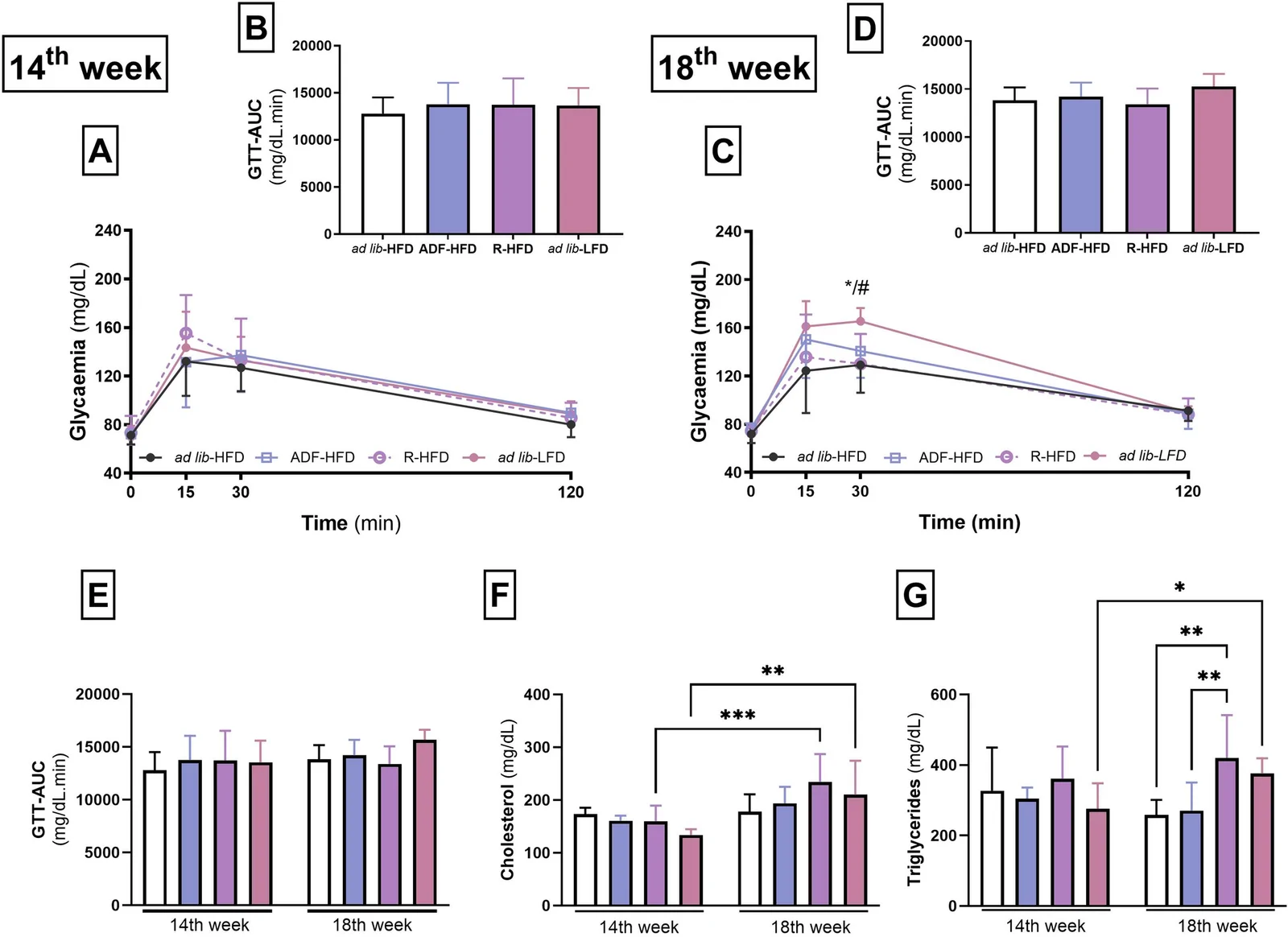
R-HFD and *ad lib-*LFD predispose animals to metabolic impairment under obesogenic diet. (**A**) GTT on 14th week (RM Two-way ANOVA: Time: F _1.468, 41.11_ = 141.9, *P* < 0.0001, Intervention: F _3, 28_ = 0.394, *P* = 0.757; Time vs. intervention: F _4.404, 41.11_ = 1.222, *P* = 0.316). (**B**) GTT-AUC on the 14th week (One-way ANOVA: F _2, 23_ = 0.468, *P* = 0.631). **(C)** GTT on 18th week (RM Two-way ANOVA: Time: F _1.271, 34.31_ = 104.1, *P* < 0.0001, Intervention: F _3, 27_ = 2.508, *P* = 0.080, Time vs. intervention: F _3.812, 34.31_ = 1.765, *P* = 0.160). Tukey’s post-test identified that *ad lib*-LFD had higher glycemia at 30 min than *ad lib*-HFD (* *P* < 0.05) and R-CCR (# *P* < 0.05). (**D**) GTT-AUC on the 18th week (One-way ANOVA: F _3, 28_ = 2.080, *P* = 0.125). (**E**) GTT-AUC during the experiment (RM Two-way ANOVA: Time: F _1, 54_ = 2.707, *P* = 0.105, Intervention: F _3, 54_ = 1.108, *P* = 0.354, Time vs. intervention: F _3, 54_ = 0.972, *P* = 0.412). (**F**) Cholesterol levels during the experiment (RM Two-way ANOVA: Time: F _1, 21_ = 17.91, *P* = 0.0004, Intervention: F _3, 21_ = 1.482, *P* = 0.248, Time vs. intervention: F _3, 21_ = 2.381, *P* = 0.098). (**G**) Triglycerides levels during the experiment (RM Two-way ANOVA: Time: F _1, 22_ = 0.479, *P* = 0.496, Intervention: F _3, 22_ = 3.490, *P* = 0.032, Time vs. intervention: F _3, 22_= 3.551, *P* = 0.031). Data expressed as mean ± SD. *P < 0.05, **P < 0.01, ***P < 0.001 represent differences between experimental groups (Sidak’s post-test)

None of the interventions altered the response to glucose overload at the week 14 (Fig. 3A–B). However, despite no differences in the final weight gain (18th week) (Fig. 2A), prior DR increased susceptibility to further deterioration of glycemic control once animals returned to free access to obesogenic diet (third phase). By week 18, *ad lib*-LFD rats displayed a delayed response to the glucose challenge compared with *ad lib*-HFD, a difference not captured by the GTT-AUC alone (Fig. 3C–D). When trajectories were analyzed longitudinally, paired comparisons revealed no significant increase in GTT-AUC from the second to the third phase in any groups (Fig. 3E). Fasting glycemia remained unchanged across groups, whereas *ad lib*-LFD and R-HFD produced a modest reduction in insulin levels at the end of the experiment (Table 2).

Similarly, none of the dietary interventions altered total cholesterol (Fig. 3F) or triglyceride levels (Fig. 3G) at week 14. By week 18, R-HFD displayed higher triglycerides than both *ad lib*-HFD and ADF-HFD. Paired comparisons were performed and showed that upon returning to *ad libitum* HFD (in the third phase), both R-HFD and *ad lib*-LFD exhibited increased cholesterol levels, and *ad lib*-LFD showed elevated triglycerides compared to themselves in the second phase (Fig. 3F-G). No other biochemical parameters differed among groups at the end of the experimental design (Table 2).

**Table 2:** Effects of dietary regimens on blood biochemistry after weight regain (third phase)

	ad lib-HFD	ADF-HFD	*R*-HFD	ad lib-LFD	One-way ANOVA
Glycaemia (mg/dL)	136.0 ± 12.4	129.8 ± 11.5	122.3 ± 13.0	128.2 ± 14.2	F_3, 28_ = 1.661, *P* = 0.198
Insulinemia (µU/L)	0.24 ± 0.05	0.25 ± 0.05	0.20 ± 0	0.20 ± 0	*P* = 0.053
LDL-C (mg/dL)	85.30 ± 34.53	89.47 ± 33.06	96.18 ± 63.78	97.53 ± 63.01	F_3, 28_ = 0.101, *P* = 0.958
HDL-C(mg/dL)	47.75 ± 18.48	53.56 ± 17.39	53.78 ± 27.86	38.33 ± 16.07	F_3, 28_ = 0.822, *P* = 0.492
TG to HDL ratio	8.71 ± 7.8	6.73 ± 3.9	10.78 ± 8.5	13.02 ± 9.8	F_3, 28_ = 0.946, *P* = 0.431
Total cholesterol to HDL-C	5.44 ± 4.7	4.41 ± 2.4	6.98 ± 8.3	7.52 ± 5.8	F_3, 28_ = 0.480, *P* = 0.698
LDL to HDL ratio	2.67 ± 3.2	2.07 ± 1.8	3.83 ± 6.8	3.92 ± 3.9	F_3, 28_ = 0.335, *P* = 0.799
ALT (U/L)	34.63 ± 21.78	26.00 ± 12.94	45.89 ± 21.30	30.00 ± 7.48	F_3, 28_ = 2.112, *P* = 0.121
AST (U/L)	31.50 ± 11.92	29.44 ± 20.97	42.22 ± 21.25	27.50 ± 6.74	F_3, 28_ = 1.204, *P* = 0.326
ALT to AST ratio	1.25 ± 0.96	1.25 ± 0.70	0.99 ± 0.57	0.92 ± 0.09	F_3, 28_ = 0.480, *P* = 0.698
GGT (U/L)	2.600 ± 2.20	5.556 ± 7.45	5.500 ± 5.69	4.567 ± 3.35	F_3, 28_ = 0.558, *P* = 0.646

Data expressed as mean ± SD and compared through One-way ANOVA, except insulin levels that were compared through Kruskal-Wallis

Moreover, weight rebound (during the third phase) was directly associated with LDL levels and tended to inversely correlate with HDL (*P* = 0.053) in the R-HFD group (Figure S5A-B). It was also directly associated with final triglycerides blood levels and glycemia in the *ad lib*-LFD group (Figure S5A, C), supporting that the extent of regain was closely associated with the severity of metabolic dysfunction.

### Prior CCR and DR reduce liver cellular defense after weight regain

**Fig. 4:**
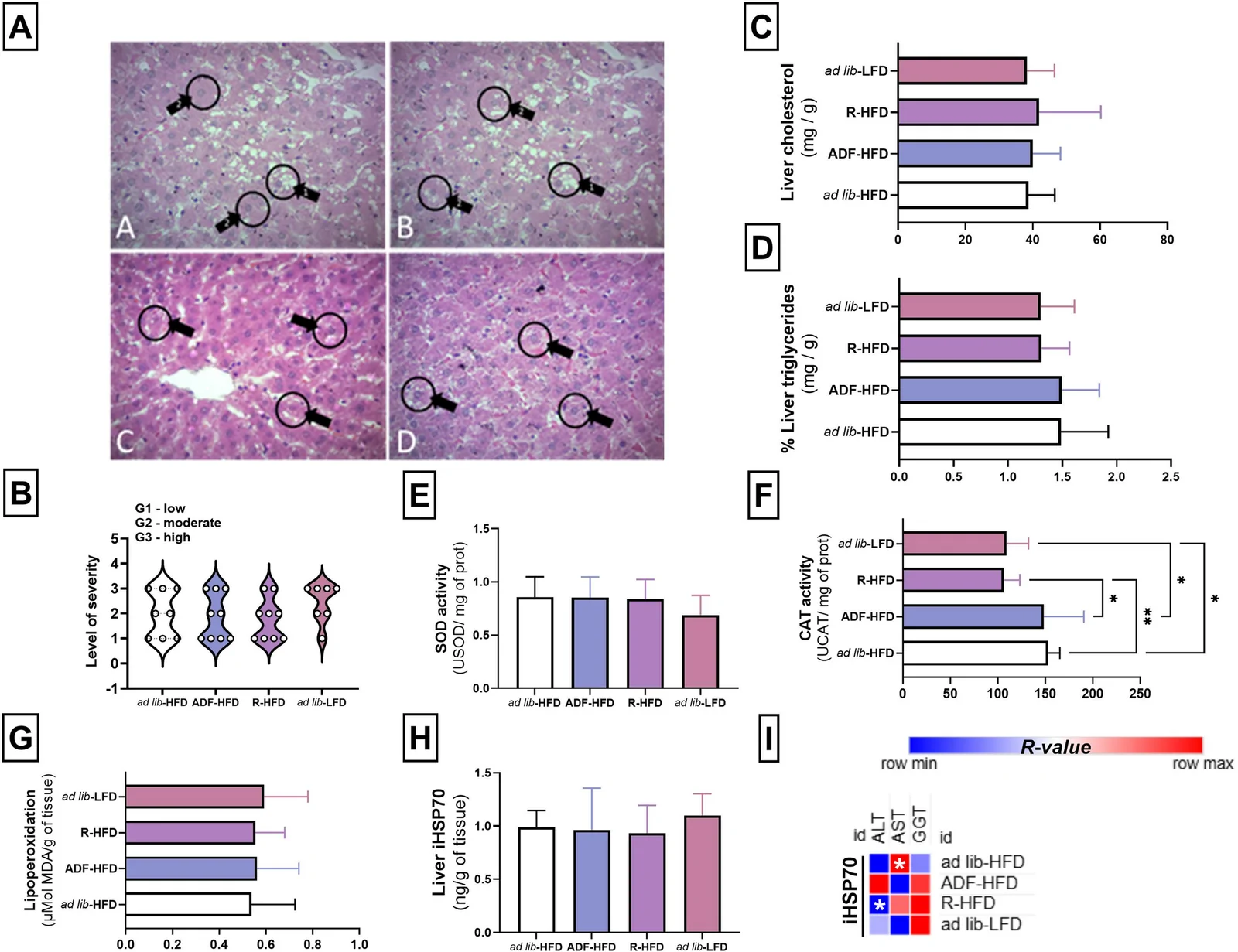
R-HFD and *ad lib-*LFD reduce liver cellular defense after weight regain. (**A**) Liver histology, H&E staining. Image *A* corresponds to *ad lib*-HFD, *B* to ADF-HFD, *C* to R-HFD and *D* to *ad lib*-LFD group. Arrows with 1 and 2 indicate macro and microvesicular damage, respectively. (**B**) Representative statistical analysis from image A (Kruskal-Wallis: *P* = 0.483). (**C**) Liver cholesterol (One-way ANOVA: F _3, 28_ = 0.146, *P* = 0.931). (**D**) Liver triglycerides (One-way ANOVA: F _3, 28_ = 0.778, *P* = 0.516). (**E**) SOD activity (One-way ANOVA: F _3, 28_ = 1.171, *P* = 0.338). F CAT activity (One-way ANOVA: F _3, 28_ = 6.894, *P* = 0.001). **G**. Lipoperoxidation (One-way ANOVA: F _3, 28_ = 0.118, *P* = 0.948). **H**. iHSP70 (One-way ANOVA: F _3, 27_ = 0.403, *P* = 0.751). **I**. Pearson’s correlations matrix between hepatic iHSP70 and enzymes. Data expressed as mean ± SD. Data expressed as mean ± SD. *P < 0.05 and **P < 0.01 represent differences between experimental groups (Tukey’s post-test)

In the end of the third phase, all experimental groups presented histological features of liver steatosis, with lipid deposition, hepatocytes ballooning, but absence of fibrosis and chronic inflammatory features (Fig. 4A). The severity was considered similar among the groups (Fig. 4B), consistent with the absence of changes in hepatic cholesterol (Fig. 4C) or triglyceride content (Fig. 4D).

Based on similar histological profiles, we next examined whether the dietary interventions produced subclinical alterations in hepatic or systemic stress responses. In the liver, R-HFD and *ad lib*-LFD groups had reduced CAT activity (Fig. 4F), whereas SOD activity (Fig. 4E) and lipid peroxidation (Fig. 4G) remained unchanged. Hepatic iHSP70 levels were also similar across groups (Fig. 4H); however, they correlated positively with AST in *ad lib*-LFD animals and inversely with ALT in the R-HFD group (Fig. 4I), suggesting context-dependent associations between HSR modulation and hepatocellular stress.

### Prior CCR and DR reduce eWAT cytoprotection after weight regain

**Fig. 5:**
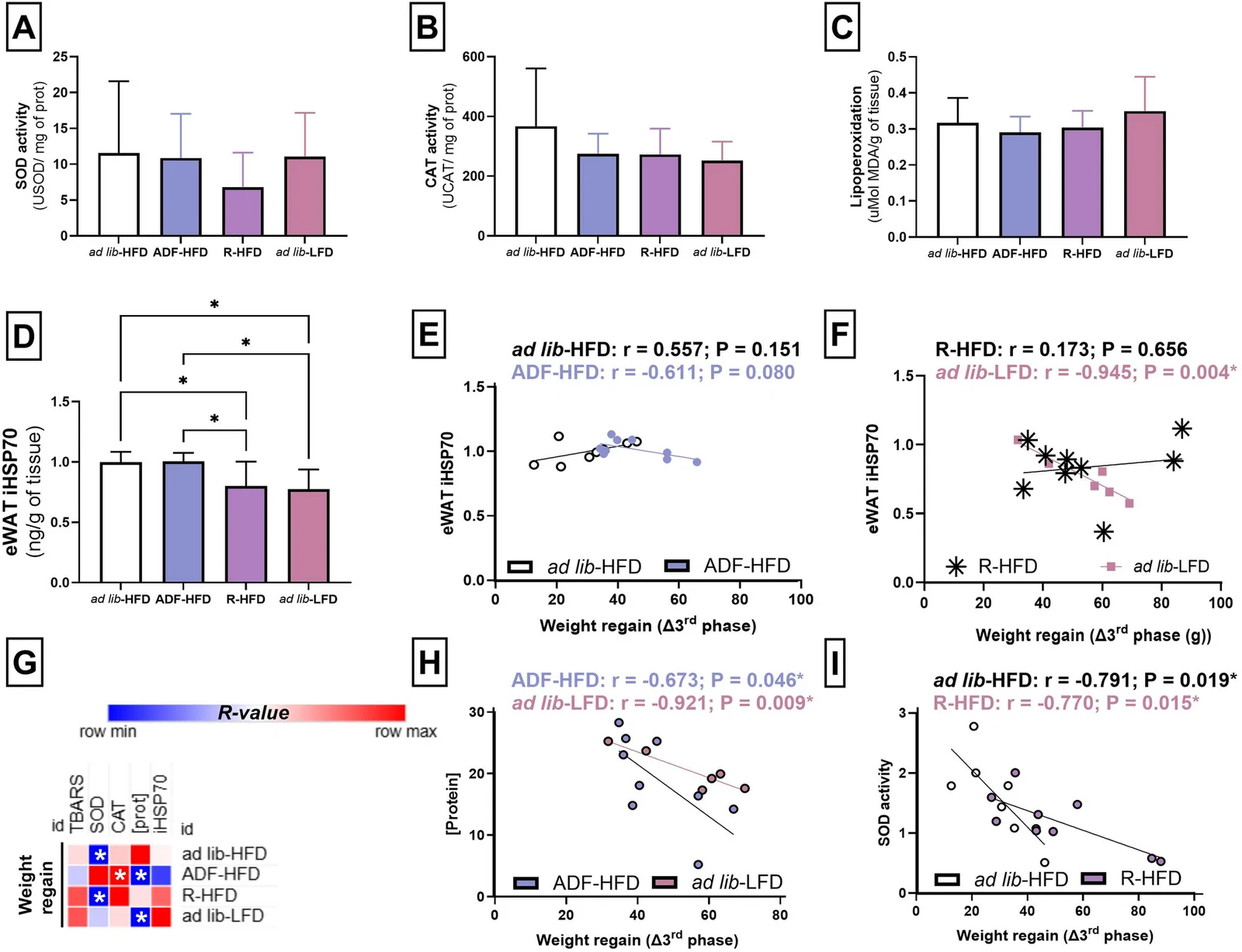
Dietary regimes affect eWAT (**A**-**F**) and gastrocnemius muscle (**G**-**I**) after weight regain. (**A**) SOD activity (One-way ANOVA: F _3, 28_ = 0.855, *P* = 0.475). (**B**) CAT activity (One-way ANOVA: F _3, 28_ = 1.503, *P* = 0.235). (**C**) Lipoperoxidation (One-way ANOVA: F _3, 28_ = 1.111, *P* = 0.361). (**D**) iHSP70 (One-way ANOVA: F _3, 27_ = 6.186, *P* = 0.002). E-F. Pearson’s correlation between eWAT iHSP70 (Fold-change) and weight regain (g) in the *ad lib*-HFD and ADF-HFD groups (**E**) and R-HFD and *ad lib-*LFD groups (**F**). **G**. Pearson’s correlation matrix between weight regain (g) and gastrocnemius redox parameters. **H**-**I**. Pearson’s correlation between weight regain (g) and muscle protein concentration (mg/mL) (**H**) and SOD activity (USOD/mg prot) (**I**). Data expressed as mean ± SD. *P < 0.05 represent differences between experimental groups (Tukey’s post-test)

HFD consumption and obesity decrease HSR [23], while contributes to systemic inflammation [28]. In our study, we did not detect any differences in the eWAT’s redox profile among groups at the end of the third phase (Fig. 5A–C). However, R-HFD and *ad lib*-LFD presented reduced iHSP70 content in this depot (Fig. 5D). Intra-group analyses revealed meaningful associations. Weight regain (throughout the third phase) tended to inversely associate with eWAT’s iHSP70 levels in the ADF-HFD group (Fig. 5E), and in the *ad lib*-LFD group (Fig. 5F).

In the gastrocnemius, none of the oxidative or inflammatory parameters were affected by the interventions (Table S2). Intra-group analyses, however, revealed meaningful associations. Weight regain was inversely correlated with total protein content in the ADF-HFD and *ad lib*-LFD groups (Fig. 5G–H), positively correlated with CAT activity in ADF-HFD (*r* = 0.758, *P* = 0.017) (Fig. 5G), and negatively correlated with SOD in the *ad lib*-HFD and R-HFD groups (Fig. 5G, I). These relationships suggest that the metabolic context of weight regain modulates tissue-specific stress responses differently depending on the preceding dietary strategy.

### CCR and DR improve leukocytes inflammatory indexes after weight regain

**Fig. 6:**
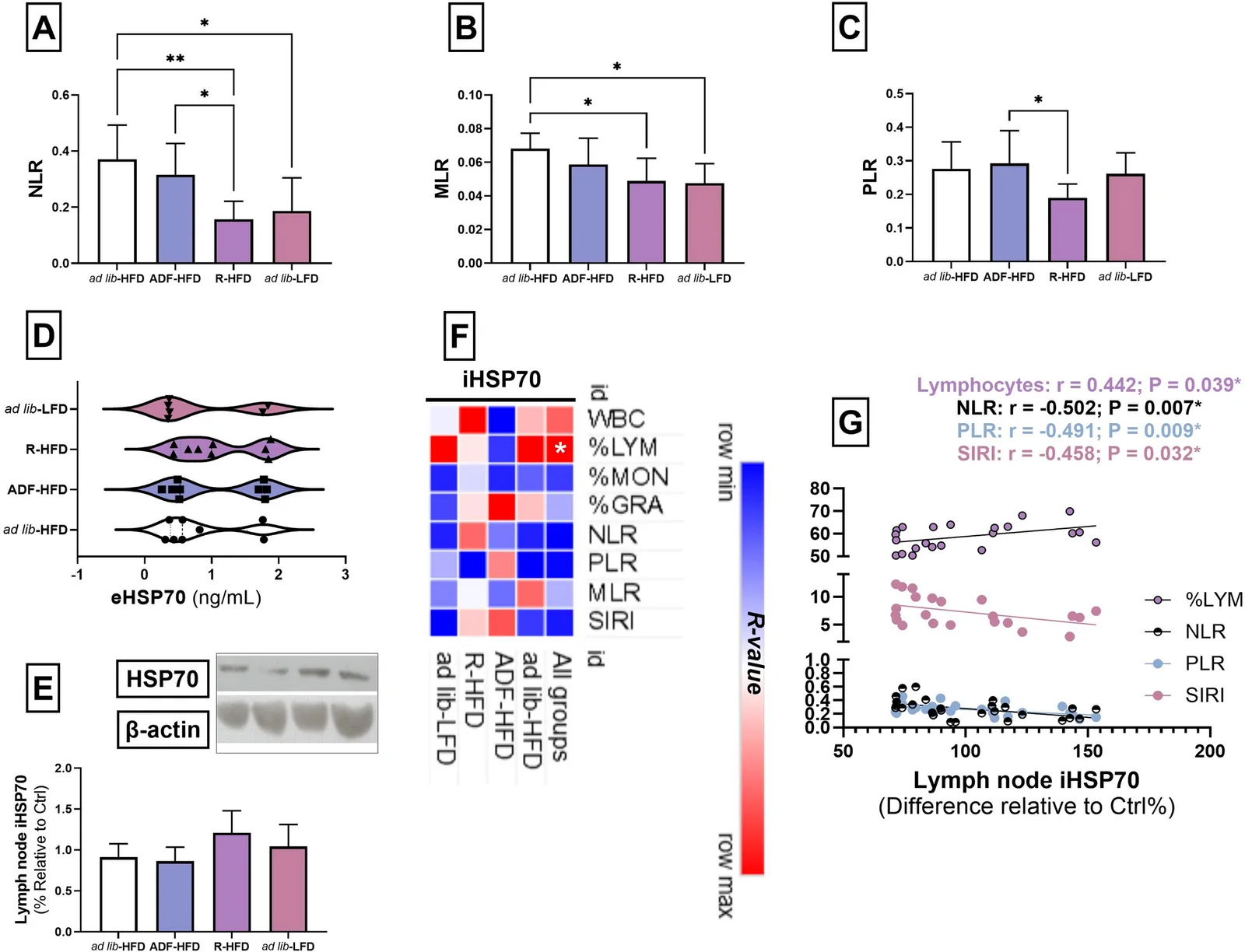
R-HFD and *ad lib-*LFD attenuate markers of systemic inflammation after weight regain. (**A**) Neutrophils to lymphocytes ratio (NLR) (One-way ANOVA: F 3, 28 = 7.756, *P* = 0.0006). (**B**) Monocytes to lymphocytes ratio (MLR) (One-way ANOVA: F _3, 28_ = 4.239, *P* = 0.013). (**C**) Platelets to Lymphocytes ratio (PLR) (One-way ANOVA: F _3, 28_ = 3.292, *P* = 0.035). (**D**) eHSP70 (One-way ANOVA: F _3, 28_ = 0.342, *P* = 0.795). (**E**) Lymph node iHSP70 (One-way ANOVA: F _3, 22_ = 3.246, *P* = 0.041). (**F**) Pearson’s correlation matrix between lymph node iHSP70 and immune parameters. (**G**) Pearson’s correlation analysis between iHSP70 (% relative to *ad lib*-HFD), lymphocytes (%), NLR, PLR and SIRI considering all groups together. Data expressed as mean ± SD. *P < 0.05 and ** *P* < 0.01 represent differences between experimental groups (Tukey’s post-test)

Given the central role of adipose tissue in propagating inflammation during obesity and insulin resistance, we assessed hematological markers of systemic inflammation at the end of third phase. Both R-HFD and *ad lib*-LFD reduced the percentage of neutrophils and increased lymphocyte proportion (Table 3). Consistently, these groups showed lower NLR (Fig. 6A) and MLR (Fig. 6B). R-HFD also exhibited a reduced PLR compared with ADF-HFD (Fig. 6C). None of the interventions altered circulating eHSP70 levels (Fig. 6D). Nonetheless, R-HFD enhanced lymph nodes iHSP70 expression (Fig. 6E, S6), which correlated positively with lymphocyte percentage and inversely with NLR, PLR, and SIRI (Fig. 6F–G), suggesting a link between local HSR modulation and systemic inflammatory tone.

**Table 3:** CCR and DR improve leukocytes inflammatory indexes after weight regain

	ad lib-HFD	ADF-HFD	*R*-HFD	ad lib-LFD	One-way ANOVA
WBC (/mm^3^)	4125 ± 1176	4367 ± 1455	5800 ± 1749	4100 ± 1149	F_3, 28_ = 2.692, *P* = 0.065
Neutrophils (%)	25 ± 6	22 ± 6	13 ± 4*/**	14 ± 8*	F_3, 28_ = 9.256, *P* = 0.0002
Lymphocytes (%)	70 ± 6	73 ± 6	83 ± 5*/**	81 ± 8*	F_3, 28_ = 8.652, *P* = 0.0003
Monocytes (%)	5 ± 1	4 ± 1	4.0 ± 1	4 ± 1	F_3, 28_ = 1.868, *P* = 0.158
SIRI	8.14 ± 2.44	7.87 ± 2.49	5.08 ± 1.49	7.14 ± 3.37	F_3, 23_ = 2.856, *P* = 0.059
Platelets (0.10^3^/µL)	743.3 ± 143.1	843.3 ± 91.74	854.0 ± 50.20	813.7 ± 71.42	F_3, 28_ = 2.261, *P* = 0.103

Data expressed as mean ± SEM, and compared through One-way ANOVA, followed by Tukey. * *P* < 0.05 *vs. ad lib*-HFD, ** vs. ADF-HFD. 

## Discussion

Weight loss remains one of the most effective strategies for preventing chronic diseases. Yet nearly half of individuals eventually regain the weight they lose, making long‑term weight maintenance a major challenge. In this study, we showed that ADF, CCR, and DR all slowed weight gain in growing rats, but stopping these interventions led to rapid weight rebound on an obesogenic diet. Among the three approaches, CCR, and DR increased susceptibility to glucose intolerance, hypercholesterolemia, and hypertriglyceridemia under *ad libitum* HFD feeding, and they weakened hepatic and WAT cellular defenses. Interestingly, both interventions also reduced peripheral inflammatory markers, which were inversely proportional to lymph node cell responses. Overall, our findings indicate that the greater weight regain after stopping dietary strategies produced metabolic disturbances and impaired redox homeostasis. In contrast, ADF did not produce harmful metabolic effects, suggesting it may offer a more protective approach for mitigating obesity‑related complications.

IF has gained prominence both for cultural and religious reasons and randomized clinical trials have shown that it produces weight loss comparable to CCR [16]. Nevertheless, varying degrees of weight regain occur after discontinuing any of these dietary strategies [6]. To better investigate this issue, we structured our experiment into three phases, weight gain, weight loss (or delay in weight gain), and maintenance/regain, using a well-established model of diet-induced obesity and glucose intolerance as the baseline [27–31, 41]. Paired comparisons confirmed that the initial gain phase was successful, and that animals were obese and glucose intolerant prior to the interventions. As expected, all dietary approaches slowed weight gain during the second phase but increased susceptibility to rebound once animals returned to free access to an obesogenic diet in the maintenance/regain (third) phase. Although the groups did not differ in final body weight or in the Lee Index, within-group analyses revealed that all interventions reduced the Lee Index during the second phase, except CCR. Notably, CCR was the only intervention associated with an increase in this index during the third phase. These early findings support the conclusion that CCR produced the most pronounced weight rebound.

Analyses of energetic efficiency have suggested that IF may offer an advantage over CCR in promoting weight loss relative to energy intake in mice [56]. Consistent with this, the second phase of our experiment showed a marked reduction in energy intake in both the ADF-HFD and R-HFD groups, with R-HFD exhibiting the lowest intake. Interestingly, during the maintenance/regain (third) phase, animals in the R-HFD and ADF-HFD groups appeared to consume more food when returned to an obesogenic diet, although their average intake remained lower than that of the *ad lib*-HFD group. In contrast, the *ad lib*-LFD group displayed the highest energy intake in both phases compared to control, which received an HFD *ad libitum* throughout the experiment. This pattern of intake may reflect underlying neurological regulation. Chronic high-fat feeding disrupts leptin-mediated activation of proopiomelanocortin (POMC) neurons [57], impairing satiety signaling even when animals are subsequently offered balanced chow. Conversely, CCR [33, 56] and ADF [58] enhance hypothalamic POMC expression and reduce resting energy expenditure [16], thereby lowering the amount of food required to maintain body weight. Once animals return to access an obesogenic diet *ad libitum*, this POMC regulation may again become impaired, providing a plausible explanation for the weight rebound observed across the experiment.

Body-weight fluctuation during “yo-yo dieting” has been associated with a higher risk of diabetes compared with stable obesity in men [12]. Given the pronounced weight rebound observed in animals previously subjected to dietary interventions, we next examined their metabolic responses. Immediately after the second phase, glucose tolerance and circulating cholesterol and triglyceride levels remained unchanged in our obesity model. Likewise, despite its beneficial effects on body weight, ADF did not alter glycemic nor lipid profiles in middle-aged female rats [55]. In contrast, prior DR (*ad lib*-LFD intake) increased susceptibility to glucose intolerance when animals had free access to an obesogenic diet, as evidenced by delayed restoration of glucose homeostasis and by reduced insulin levels, a trend also observed in the R-HFD group at the end of the third phase. These same interventions predisposed animals to hypercholesterolemia and hypertriglyceridemia after weight regain (third phase). The metabolic disturbances accompanying weight regain after CCR and DR mirrored previously described oscillations in malic enzyme, pyruvate kinase, fatty acid synthase, and acetyl-CoA carboxylase activities, culminating in increased *de novo* fatty acid synthesis during weight cycling [15]. Notably, the dietary strategies that produced the most pronounced alterations in lipid profiles were also those associated with the greatest fluctuations in body weight and Lee Index under an obesogenic diet. Although initially unexpected, these findings align with evidence that weight variability is linked to higher mortality and increased cardiovascular events, independent of traditional risk factors [13].

Long-term exposure to this HFD is known to induce hepatic steatosis [29]. In line with this, all experimental groups exhibited lipid accumulation, hepatocyte ballooning and comparable lesion severity, although no fibrosis or features of chronic inflammation were detected. Similarly, the plasmatic levels of hepatic enzymes and hepatic lipoperoxidation were not affected by the interventions. However, CAT activity was dropped by CCR and DR, potentially increasing susceptibility to oxidative injury and dysfunction. Previous work has shown that moderate caloric restriction (8–25%) over comparable or longer durations can attenuate protein oxidative damage [32, 59] and enhances glucose-6-phosphate dehydrogenase activity, thereby supporting glutathione’s recycling [32]. Therefore, these findings suggest that the impaired antioxidant defense observed in the liver was not attributable to the interventions themselves but conditioned to the return to *ad libitum* obesogenic diet after the dramatic weight loss caused by them.

Prolonged alterations in fatty acid metabolism during weight cycling affect both adipose depots and the liver, although hepatic changes tend to be less pronounced than those observed in WAT [15]. In our study, final eWAT mass did not differ among groups; however, previous work has shown that epididymal adipocytes can be up to 80% larger in weight-cycling animals than in weight-stable obese controls [15]. Such hypertrophy contributes to metabolic dysfunction and systemic inflammation [14]. Despite these metabolic disturbances, the prior dietary interventions did not modify the eWAT redox profile upon return to obesogenic diet (third phase). In contrast, both R-HFD and *ad lib*-LFD groups exhibited reduced iHSP70 content in this depot. Ex vivo studies have demonstrated that stress in subcutaneous adipocytes during weight-loss interventions is closely linked to subsequent weight regain, with weight-lowering strategies increasing HSP70 and HSP60—proteins proposed to play key roles in post-diet weight regain [60]. Our focus, however, was on a visceral adipose depot, which is more strongly implicated in metabolic dysfunction and inflammation [61]. Previous work from our group and collaborators has shown that HFD consumption increases iHSP70 expression in eWAT [27] until adipocytes eventually lose the capacity to mount an adequate HSR, entering a chronic inflammatory state associated with insulin resistance and steatosis severity [23]. Supporting this relationship, hepatic iHSP70 correlated positively with AST levels in the *ad lib*-HFD group and inversely with ALT in the R-HFD group, while eWAT iHSP70 was inversely associated with weight regain, particularly in the *ad lib*-LFD group.

Studies have shown that weight recovery/oscillation is associated with tissue-specific insulin resistance, marked by a preference for storage via *de novo* lipogenesis and muscular insulin resistance [62]. Given the metabolic impairments specially verified after CCR and DR, we analyzed gastrocnemius redox homeostasis. Although we did not observe any differences in these parameters among the experimental groups, weight regain positively correlated with SOD in *ad lib*-HFD and ADF-HFD, and with catalase in the last group. In parallel, it was inversely correlated with protein concentration in ADF-HFD, R-HFD and *ad lib*-LFD groups, and with SOD in the last group mentioned, showing that weight regain had more effect on gastrocnemius redox parameters than the dietary interventions per se. Considering that DR and CCR allowed the highest weight rebound, the associations were more observed among these groups, suggesting a subtle trend to jeopardize muscle composition and redox profile. Previous histological analysis identified that, despite no difference in the gross aspects of the skeletal muscle, intermittent fasting and exercise exhibits a certain degree of protection [10], corroborating the metabolic and cellular protection observed in our study. Animals undergoing DR or CCR, on the other hand, become more susceptible to the metabolic, redox and impaired muscle health caused by obesogenic diet.

Despite promoting weight loss, simple DR does not reverse the underlying biological dysfunctions associated with obesity in mice [10]. In contrast, CCR and IF have consistently been shown to reduce circulating inflammatory markers [10, 63] without compromising essential cell-mediated immunity [63]. In our study, CCR and DR produced a notable shift in leukocyte subtype distribution. Both interventions increased circulating lymphocytes and reduced neutrophil proportions, resulting in lower NLR, MLR, and PLR values. Supporting this coordinated immunological response, mesenteric lymph nodes, which integrate peripheral and adipose tissue immune signals, displayed elevated iHSP70 expression in the R-HFD group. This increase correlated positively with lymphocyte abundance and negatively with NLR and PLR. This leukocyte remodeling within an obese background underscores the role of immune cells, particularly CD4⁺ T cells, in sustaining the so-called “obesity memory,” a mechanism that predisposes animals to accelerated weight regain [64]. During rebound weight gain, the decline in eWAT iHSP70 levels observed in R-HFD- and *ad lib-*LFD-treated animals reflects adipocyte exhaustion and may signal the onset of senescence or cell death. As adipocytes fail to resolve their initial stress, they transition into a progressively inflammatory state and recruit additional immune cells for clearance [22].

Our findings demonstrate that weight regain, particularly following CCR and DR, heightens susceptibility to metabolic, oxidative, and immune dysfunction when animals return to an obesogenic diet. Although our study did not include proteomic analysis to further dissect the mechanisms underlying these alterations, the results provide a reliable early signal that warrants deeper investigation by future research groups. Consistent with clinical evidence showing comparable weight-loss efficacy between IF and CCR [6], our data reveal an important distinction: ADF uniquely protected against the metabolic and oxidative disturbances that emerged during weight rebound. Furthermore, our results emphasize that longer-term interventions may be more manageable and beneficial, as they tend to produce less pronounced weight regain and thereby reduce the associated physiological consequences [6].

## Conclusion

All interventions tested were effective in delaying weight gain and improving body composition indexes; however, discontinuing any of them led to rapid weight regain when returned to an obesogenic diet. Our findings indicate that, despite this rebound, ADF does not trigger adverse metabolic outcomes and may provide a more favorable metabolic profile regarding obesity-related complications. In contrast, under renewed HFD *ad libitum* consumption, DR and CCR, in comparison to ADF, may have increased susceptibility to glucose intolerance, as suggested by an elevation in plasma glucose level in the GTT, and to an increase in plasma triglyceride levels. These approaches also impaired hepatic and eWAT antioxidant and anti-inflammatory responses and heightened lymph node stress. Notably, the extent of weight regain was closely associated with the severity of metabolic and immune-inflammatory profile. These findings suggest that, despite similar weight-loss efficacy, ADF may better preserve metabolic homeostasis during weight regain compared with CCR and DR.

## Supplementary Information

Below is the link to the electronic supplementary material.

Supplementary Material 1

Supplementary Material 2

## Data Availability

The data will be available upon reasonable request.

## Declarations

### Declaration of AI use

ChatGPT (OpenAI) was employed for grammar verification and minor language refinement. The scientific content, data interpretation, information structure and conclusions were entirely made by the authors.
