# Supplementary material for: Continuous caloric restriction and diet replacement but not alternate-day fasting may enhance susceptibility to glucose intolerance, dyslipidemia and cell stress under renewed high-fat diet
Source: J Diabetes Metab Disord. 2026 Oct 21;25(2):293. doi: 10.1007/s40200-026-02112-1 (PMC13687523; doi:10.1007/s40200-026-02112-1)
Supplement: Supplementary file 1 [file 40200_2026_2112_MOESM1_ESM.docx]

**Continuous caloric restriction and diet replacement but not alternate-day fasting may enhance susceptibility to glucose intolerance, dyslipidemia and cell stress under renewed high-fat diet**

Andrea Cristiani Beuren^1*^, Jaíne Borges dos Santos^1*^, Natascha Fill Izolan^1*^, Lílian Correa Costa-Beber^1,2*/**^, Lucas Machado Sulzbacher^1^, Matias Nunes Frizzo^1^, Pauline Brendler Goettems-Fiorin^1^, Thiago Gomes Heck^1,3,4^, Mirna Stela Ludwig^1,4^

**SUPPLEMENTARY MATERIAL**

**Extended results**

**Figure S1: HFD consumption *ad libitum* promotes glucose intolerance. A.** GTT at the baseline and at the end of the first phase (8^th^ week). One-way ANOVA with repeated measures: Time (min): F _2.092, 129.7_ = 386.8, P < 0.0001. Start *vs* 8^th^ week: F _1, 62_ = 1.913, P = 0.171. Interaction between factors: F _2.092, 129.7_ = 10.53, P < 0.0001. **B.** First *vs* final GTT-AUC. Paired T-test: **P = 0.002 *vs* Start. Data expressed as mean ± SEM.

**Figure S2: HFD consumption induces obesity and glucose intolerance.** Male 12 weeks-old Wistar rats were divided into two groups, *ad lib*-LFD (n = 24) or *ad lib-*HFD (n = 26) receiving standard or HFD, uninterruptedly, for eight weeks (CEUA 001/2015). **A.** Body weight during the experiment. RM Two-way ANOVA: Time: F _12, 507_ = 72.41, P < 0.0001. Diet: F _1, 507_ = 44.12, P < 0.0001. Time *vs* Diet: F _12, 507_ = 2.013, P = 0.021. **B.** GTT curve on the 4^th^ week. RM Two-way ANOVA: Time: F _1.279, 61.37_ = 250.0, P < 0.0001. Diet: F _1, 48_ = 25.40, P < 0.0001. Time *vs* Diet: F _1.279, 61.37_ = 2.441, P = 0.115. **C.** GTT-AUC on the 4^th^ week. T-test: P < 0.0001. **D.** GTT curve on the 8^th^ week. RM Two-way ANOVA: Time: F _1.213, 58.21_ = 222.5, P < 0.0001. Diet: F _1, 48_ = 49.11, P < 0.0001. Time *vs* Diet: F _1.213, 58.21_ = 1.970, P = 0.163. **E.** GTT-AUC on the 8^th^ week. T-test: P < 0.0001. Data expressed as mean ± SEM. Differences between groups were represented as * P ˂ 0.05, **P ˂ 0.01, *** P ˂ 0.001 and **** P ˂ 0.0001 *vs* *ad lib*-LFD.

**Figure S3: Only *ad lib*-HFD group presented an increase in animals’ length during the second phase (14^th^ week).** Data expressed in mean ± SD. RM Paired analysis identified that *ad lib*-HFD, ADF-HFD and *ad lib*-LFD enhanced their NAL along the second phase (intervention) (RM Two-way ANOVA: Time: F _1, 28_ = 50.25, P < 0.0001. Intervention: F _3, 28_ = 0.058, P = 0.980. Time *vs* Intervention: F _3, 28_ = 2.859, P = 0.054). No differences among groups were identified.

**Figure S4:** **Effects of different dietary interventions on food consumption. A.** AUC-energetic intake during the intervention (second) phase (One-way ANOVA: F _3, 28_ = 346.6, P < 0.0001). **B.** AUC-energetic intake during the regain/maintenance (third) phase (One-way ANOVA: F _3, 28_ = 57.45, P < 0.0001). **C.** Representative consumption (Kcal/animal/week) throughout the experiment. There was no obvious spillage, judged by looking, on any day. Data expressed in mean ± SD. ***P ˂ 0.001 and ****P ˂ 0.0001 represent differences between experimental groups (Tukey’s post-test).

**Figure S5: Weight regain in the third phase correlates with final metabolic impairment in R-HFD and *ad lib*-LFD groups. A.** Pearson’s correlations matrix between weight regain (g) and metabolic parameters. **B.** Pearson’s correlations between weight regain (g), final HDL (mg/dL) and LDL (mg/dL) in the R-HFD group**. C.** Pearson’s correlations between weight regain, final triglycerides and glycemia in the *ad lib*-LFD group.

**Table S1:** Studies used in sample size calculation.

| **Reference** | **Number of animals per experimental group** | **Dietary intervention** | **Period of application** |
| --- | --- | --- | --- |
| (Higashida et al., 2013) | 12 | ADF | 6 |
| (Manzanero et al., 2014) | 10 | TRF | 12 |
| (Seimon et al., 2016) | 10 | MFR | 12 |
| (Gotthardt et al., 2016) | 8 | ADF | 8 |
| (Gotthardt & Bello, 2017) | 7 | ADF | 4 |
| (Delahaye et al., 2018) | 10 | TRF | 8 |
| (Wilson et al., 2018) | 8 to 10 | MFR | 12 |

Notes: ADF = *Alternate Day Fasting*; MFR = *Modified Fasting Regimens*; TRF = *Time-Restricted Feeding*.

**Table S2: Dietary regimes do not affect gastrocnemius oxidative and inflammatory response after weight rebound (third phase).**

| **Parameter** | ***ad lib*-HFD** | **ADF-HFD** | **R-HFD** | ***ad lib*-LFD** | **One-way ANOVA** |
| --- | --- | --- | --- | --- | --- |
| **Protein concentration**  (mg/mL) | 17.09 ± 3.251 | 19.01 ± 7.282 | 19.11 ± 3.307 | 20.11 ± 3.205 | F_3, 28_ = 0.511, P = 0.677 |
| **SOD activity**  (USOD/mg prot) | 1.559 ± 0.692 | 1.517 ± 0.796 | 1.175 ± 0.458 | 1.114 ± 0.492 | F_3, 28_ = 0.994, P = 0.409 |
| **CAT activity** (UCAT/mg prot) | 6.273 ± 1.868 | 6.660 ± 1.708 | 6.238 ± 1.308 | 5.880 ± 1.302 | F_3, 28_ = 0.302, P = 0.823 |
| **Lipoperoxidation** (µMol MDA/g tissue) | 10.26 ± 2.776 | 9.696 ± 2.000 | 8.032 ±1.485 | 9.263 ± 1.728 | F_3, 28_ = 1.821, P = 0.166 |
| **iHSP70**  (ng/g tissue) | 0.906 ± 0.451 | 0.746 ± 0.483 | 1.018 ±0.371 | 0.971 ± 0.272 | F_3, 28_ = 0.717, P = 0.549 |

Data expressed as mean ± SD.

**REFERENCES**

Delahaye, L. B., Bloomer, R. J., Butawan, M. B., Wyman, J. M., Hill, J. L., Lee, H. W., Liu, A. C., McAllan, L., Han, J. C., & van der Merwe, M. (2018). Time-restricted feeding of a high-fat diet in male C57BL/6 mice reduces adiposity but does not protect against increased systemic inflammation. *Applied Physiology, Nutrition, and Metabolism*, *43*(10), 1033–1042. https://doi.org/10.1139/apnm-2017-0706

Gotthardt, J. D., & Bello, N. T. (2017). Meal pattern alterations associated with intermittent fasting for weight loss are normalized after high-fat diet re-feeding. *Physiology & Behavior*, *174*, 49–56. https://doi.org/10.1016/j.physbeh.2017.02.046

Gotthardt, J. D., Verpeut, J. L., Yeomans, B. L., Yang, J. A., Yasrebi, A., Roepke, T. A., & Bello, N. T. (2016). Intermittent Fasting Promotes Fat Loss With Lean Mass Retention, Increased Hypothalamic Norepinephrine Content, and Increased Neuropeptide Y Gene Expression in Diet-Induced Obese Male Mice. *Endocrinology*, *157*(2), 679–691. https://doi.org/10.1210/en.2015-1622

Higashida, K., Fujimoto, E., Higuchi, M., & Terada, S. (2013). Effects of alternate-day fasting on high-fat diet-induced insulin resistance in rat skeletal muscle. *Life Sciences*, *93*(5–6), 208–213. https://doi.org/10.1016/j.lfs.2013.06.007

Manzanero, S., Erion, J. R., Santro, T., Steyn, F. J., Chen, C., Arumugam, T. V, & Stranahan, A. M. (2014). Intermittent Fasting Attenuates Increases in Neurogenesis after Ischemia and Reperfusion and Improves Recovery. *Journal of Cerebral Blood Flow & Metabolism*, *34*(5), 897–905. https://doi.org/10.1038/jcbfm.2014.36

Seimon, R. V., Shi, Y.-C., Slack, K., Lee, K., Fernando, H. A., Nguyen, A. D., Zhang, L., Lin, S., Enriquez, R. F., Lau, J., Herzog, H., & Sainsbury, A. (2016). Intermittent Moderate Energy Restriction Improves Weight Loss Efficiency in Diet-Induced Obese Mice. *PLOS ONE*, *11*(1), e0145157. https://doi.org/10.1371/journal.pone.0145157

Wilson, R., Deasy, W., Stathis, C., Hayes, A., & Cooke, M. (2018). Intermittent Fasting with or without Exercise Prevents Weight Gain and Improves Lipids in Diet-Induced Obese Mice. *Nutrients*, *10*(3), 346. https://doi.org/10.3390/nu10030346
