# Supplementary material for: Continuous caloric restriction and diet replacement but not alternate-day fasting may enhance susceptibility to glucose intolerance, dyslipidemia and cell stress under renewed high-fat diet
Source: J Diabetes Metab Disord. 2026 Oct 21;25(2):293. doi: 10.1007/s40200-026-02112-1 (PMC13687523; doi:10.1007/s40200-026-02112-1)
Supplement: Supplementary file 2 [file 40200_2026_2112_MOESM2_ESM.docx]

**Continuous caloric restriction and diet replacement but not alternate-day fasting may enhance susceptibility to glucose intolerance, dyslipidemia and cell stress under renewed high-fat diet**

Andrea Cristiani Beuren^1*^, Jaíne Borges dos Santos^1*^, Natascha Fill Izolan^1*^, Lílian Correa Costa-Beber^1,2*/**^, Lucas Machado Sulzbacher^1^, Matias Nunes Frizzo^1^, Pauline Brendler Goettems-Fiorin^1^, Thiago Gomes Heck^1,3,4^, Mirna Stela Ludwig^1,4^

**SUPPLEMENTARY MATERIAL**

**Uncropped WB**

**Figure S6:** **Full-length lymph nodes Western blotting images corresponding to Figure 6E.** The pink square represents the chosen bands to Figure 6E.
